# Polymorphism Interaction Analysis (PIA): a method for investigating complex gene-gene interactions

**DOI:** 10.1186/1471-2105-9-146

**Published:** 2008-03-06

**Authors:** Leah E Mechanic, Brian T Luke, Julie E Goodman, Stephen J Chanock, Curtis C Harris

**Affiliations:** 1 Laboratory of Human Carcinogenesis, National Cancer Institute, NIH, Bethesda, MD, USA; 2 SAIC-Frederick, Advanced Biomedical Computing Center, National Cancer Institute, NIH, Frederick, MD, USA; 3 Gradient, Inc.; Boston, MA, USA; 4 Pediatric Oncology Branch, National Cancer Institute, Advanced Technology Center, Gaithersburg, MD, USA

## Abstract

**Background:**

The risk of common diseases is likely determined by the complex interplay between environmental and genetic factors, including single nucleotide polymorphisms (SNPs). Traditional methods of data analysis are poorly suited for detecting complex interactions due to sparseness of data in high dimensions, which often occurs when data are available for a large number of SNPs for a relatively small number of samples. Validation of associations observed using multiple methods should be implemented to minimize likelihood of false-positive associations. Moreover, high-throughput genotyping methods allow investigators to genotype thousands of SNPs at one time. Investigating associations for each individual SNP or interactions between SNPs using traditional approaches is inefficient and prone to false positives.

**Results:**

We developed the Polymorphism Interaction Analysis tool (PIA version 2.0) to include different approaches for ranking and scoring SNP combinations, to account for imbalances between case and control ratios, stratify on particular factors, and examine associations of user-defined pathways (based on SNP or gene) with case status. PIA v. 2.0 detected 2-SNP interactions as the highest ranking model 77% of the time, using simulated data sets of genetic models of interaction (minor allele frequency = 0.2; heritability = 0.01; N = 1600) generated previously [Velez DR, White BC, Motsinger AA, Bush WS, Ritchie MD, Williams SM, Moore JH: A balanced accuracy function for epistasis modeling in imbalanced datasets using multifactor dimensionality reduction. Genet Epidemiol 2007, 31:306–315.]. Interacting SNPs were detected in both balanced (20 SNPs) and imbalanced data (case:control 1:2 and 1:4, 10 SNPs) in the context of non-interacting SNPs.

**Conclusion:**

PIA v. 2.0 is a useful tool for exploring gene*gene or gene*environment interactions and identifying a small number of putative associations which may be investigated further using other statistical methods and in replication study populations.

## Background

In the absence of highly penetrant, rare, genetic mutations, the risk of common diseases, such as cancer, is likely determined by a complex interplay between several genetic and environmental factors. Common genetic variation, in the form of single nucleotide polymorphisms (SNPs), are believed to modulate cancer susceptibility [1,2]. However, only a fraction of association studies examining single loci have replicated [3,4]. One reason for the lack of replication of SNP studies, in particular when considering the complexity of pathways of carcinogenesis, may include a failure to consider complex gene*gene or gene*environment interactions [5,6]. Others include chance, poor design, insufficient power, or population stratification [7,8].

With the advent of high-density SNP arrays and genome-wide association studies, the amount of genetic data available gives researchers unprecedented opportunities to explore complexity of common diseases. Using traditional methods of data analysis, such as logistic regression modeling, it is difficult to detect complex interactions due to the sparseness of data in high dimensions [9,10]. Moreover, as the number of genetic factors being investigated increases, the number of potential interactions exponentially increases. For pair-wise interactions, the number of possible interactions is N!/[2! (N-2)!], where N is the number of SNPs or factors. Therefore, if examining 10 SNPs, there are 45 possible pair-wise interactions. Testing each interaction independently would be inefficient and subject to concerns regarding multiple comparisons. Informatic tools may be used to prioritize or select SNP interactions of interest to follow up in further study. Statistical and computational methods have not kept pace with the available data [11].

A recent review examined several different approaches for studying complex genetic interactions [12]. The authors concluded that while none of the current methods is ideal, the optimal approach is to implement several methods of analysis and validate results from each method. We hypothesized that the most efficient way to detect complex genetic interactions, for follow up in future study, is to compare results using several different methodologies. We, therefore, expanded our original program, Polymorphism Interaction Analysis (PIA version 1.0) [5], to include several approaches for ranking and scoring different SNP combinations (PIA version 2.0). In addition, PIA v. 2.0 was written to account for missing data and imbalances between case and control ratios, to stratify on particular factors, and to examine user-defined pathways. In this report, we describe the modified PIA, now known as version 2.0, and evaluate the performance of PIA v. 2.0 identifying interacting alleles in simulated and experimental data sets.

## Methods

### Algorithm

PIA v. 2.0 was developed as a method of feature selection. In a data set of a large number of SNPs or features, PIA v.2.0 can be implemented to sift through the large number of interactions to aid in the selection of SNPs for future study. We theorized that no single test or informatic approach is optimal for detecting complex interactions in all situations. Therefore, PIA v. 2.0 was intended to provide several opportunities for internal validation of observed genetic interactions by examining SNP data using multiple approaches. The program is designed to simultaneously use up to seven scoring metrics for each SNP combination (to estimate quality of SNP associations), make each metric as independent as possible, account for imbalances in the number of cases and controls, and present results for the top 100 SNP combinations for each scoring function and for an overall scaled summation of all scores. Other options included in the PIA program include the ability to incorporate pathway assignments for SNPs to explore associations of particular pathways with a phenotype and an option to allow stratified analysis of SNP associations. PIA also explores the number of times particular SNPs or SNP-pairs are observed in larger combinations of SNPs (triplets or quartets).

PIA uses a case-based exclusion for missing SNP data, i.e. only those subjects that have all SNPs (in a particular combination) identified are used in the analysis. PIA v. 2.0 is a non-parametric combinatorial method [11], meaning that all combinations of a selected number (N, 1^st^–4^th ^order) of categorical exposure variables (SNPs, haplotypes, environmental factors, race) are examined. Since SNPs represent the majority of the features examined, for the remainder of this discussion, all categorical variables, or features, to be analyzed for interactions with PIA will be denoted as SNPs.

**Figure 1:**
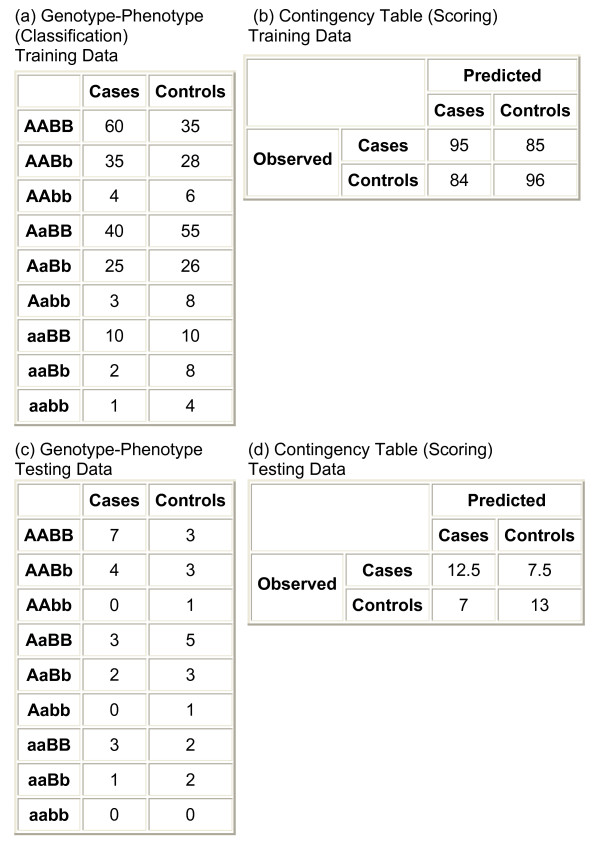
**Description of Method for Scoring Functions 1–5**. In this example, a study consists of 200 cases and 200 controls and a 10-fold cross-validation is performed. Only two SNPs are examined: A (with alleles A and a) and B (with alleles B and b) in this example. The order of samples is scrambled before training. In (a) training samples (180 cases and 180 controls) are assigned to the 9 × 2 genotype-phenotype table (classification). The genotype-phenotype table is the distribution of phenotypes (i.e. case vs. control) for all possible genotype combinations for the SNPs examined. The genotype-phenotype table is used for classification of SNPs. In this example, PIA v. 2.0 designates AABB and AABb as case-genotypes, aaBB as an undetermined-genotype, and the remaining six genotypes as control-genotypes. If the training data is selected to contribute to scoring (if the Jackknife analysis, LOO is selected), a contingency data is generated using the training data (b). The contingency table compares the observed genotype-phenotype distribution to the expected based on the genotype assignments in (a). The testing data is placed into the appropriate cells of the genotype-phenotype table (c). The contingency table for testing data (d) is generated using genotype assignments from the training data (a). Since the AABB genotype represents a case-phenotype (based on training data), the seven case samples are added to the number of true positives (NTP) and the three control samples are added to the number of false positives (NFP) in the contingency table (d). Conversely, AaBB is a control-phenotype, so the five controls are added to the number of true negatives (NTN) and the three cases are added to the number of false negatives (NFN). If a testing sample is assigned to an undetermined-phenotype (aaBB), PIA counts the assignment as half-right and half-wrong. Therefore, the three cases cause NTP and NFP to be increased by 1.5; the two controls increase NTN and NFN by 1.0. After processing all testing samples, the corresponding contingency table is shown in (d). The process is then repeated for the remaining 9 sets of testing and training samples, and all contingency tables arising from the testing samples are summed.

For a given SNP combination, the scoring first involves assigning the phenotypes (i.e. case vs. control status) for each of the possible genotypes or genotype combinations. In the case of pair-wise SNP interactions, this involves placing a sample into the appropriate cell of a 9 × 2 table (9 genotypes and 2 phenotypes) as shown in the genotype-phenotype table in Figure 1. The seven possible scoring metrics and the equations for scoring are listed in Table 1. The user may use all scoring metrics, or only specific metrics of interest.

**Table 1:** Definition of the seven scoring functions used in PIA v. 2.0.

Metric	Description	Formula^a^
1	%Correct	(NTP + NTN)/(NTP + NFN + NFP + NTN)
2	Sensitivity + Specificity	[NTP/(NTP+NFN)] + [NTN/(NFP+NTN)]
3	Positive Predictive Value (PPV)+ Negative Predictive Value (NPV)	[NTP/(NTP+NFP)] + [NTN/(NFN+NTN)]
4	Risk Ratio	[(NTP)(NFP+NTN)]/[(NFP)(NTP+NFN)]
5	Odds Ratio	[(NTP)(NTN)]/[(NFP)(NFN)]
6	Gini Index^b^	GINI_parent _- GINI_split_
		GINI(k) = 1.0 - ∑_j = 1, J _[p(j|k)]
		GINI_split _= ∑_k = 1, K _[(n_k_/n) GINI(k)]
7	Absolute Probability Difference^c^	Σ_k = 1, K _|P_1_(k) - P_2_(k)|

^a ^NTP, Number of true positives; NTN, number of true negatives; NFN, number of false negatives; NFP, number of false positives.

^b ^Gini Index is used in CART decision trees [25]. The scoring for Gini index is described under "Algorithm."

^c ^Scoring is the probability of finding a case (P_1_) in cell, k, minus the probability of observing a control (P_2_) in cell, k, summed over all the K cells in the genotype-phenotype table.

For scoring functions 1–5, an N-fold cross validation (user defined N) is implemented and this can be run multiple times. In each run, the order of the samples is scrambled before division into training and testing sets. The genotype-phenotype table for the training set is used for the assignments of genotypes. Genotype combinations with a larger number of cases than controls are considered case-genotype combinations, while combinations with more controls than cases are considered control phenotypes. When there are an equal number of cases and controls for a particular genotype combination, the combination is considered an undetermined phenotype.

After assignment of genotypes in the training samples, scoring for the first five metrics requires the formation of a 2 × 2 contingency table (Figure 1) where the number of expected cases and controls with each genotype combination (based on assignments) versus the number of cases and controls observed with a genotype combination (in the testing set, or the set used for scoring) are compared. A unique feature of PIA v. 2.0 is the handling of ties. In PIA v 2.0, for those genotype combinations that are evenly distributed among cases and controls (assigned as undetermined) in the genotype-phenotype table, the genotype combination is considered as half-right and half-wrong, i.e. count as 1/2 number of true positive (NTP) + number true negative (NTN) and 1/2 number of false positive (NFP) + number false negative (NFN) in the contingency table. This scoring procedure is repeated for the remaining sets of training and testing samples (in the example of 10-fold cross-validation, 9 additional times), and all contingency tables arising from the testing samples are summed. PIA v. 2.0 allows this scrambling and multi-fold cross-validation to be run several times; the resulting testing contingency table is the sum of all contingency tables produced by the testing samples. The more times the scrambling and multi-fold cross-validation procedure is performed, the more stable the observed associations (data not shown). In this described method for scoring functions 1–5, assignments are based on the training sets and scoring is based on the testing data using 10 runs of 10-fold cross-validation.

An alternative option in PIA v. 2.0 is to allow the training data to be included in the formation of the contingency table for scoring. This is done by performing a Jackknife (leave-one-out cross-validation, LOO) or maximum likelihood analysis of the training genotype-phenotype table. Allowing the user to include the training data was implemented as an option to increase the power for datasets with a small number of samples, as used in other programs such as MDR [10,13].

Scoring functions 6 and 7 are based on the distribution of all participants in the cells of the genotype-phenotype table. Therefore, using these functions, a cross-validation procedure is not implemented and the entire population is used. If the number of SNPs is very large, running multiple cycles of multi-fold cross-validation may be computationally prohibitive. Therefore, PIA v. 2.0 also allows a single examination of all data to be used to construct the contingency table. Therefore, a single pass through all of the data can be performed for all scoring functions.

Often in case-control studies, the case/control ratio is imbalanced, or the study consists of more controls than cases. PIA v. 2.0 includes options for accounting for imbalances in populations with particular phenotypes, or differences in case to control ratios. Using the default PIA v. 2.0 options, if the number of samples in one phenotype is significantly larger than the number of samples in the other, it is likely that all genotypes will be assigned to the more populous phenotype, resulting in biased estimates. For example, in studies with case to control ratios of 1:4, SNP combinations will be weighted towards control associations, because most genotype combinations would have a higher number of controls than cases. To circumvent this, PIA v. 2.0 allows for fractional occupations, or percents of cases/controls associated with each genotype combination, to be used in the genotype-phenotype table to determine the phenotype of each genotype. For example, if there are 90 cases and 180 controls in the training data and a genotype combination contained 12 cases and 20 controls, the fractional occupations would be 13.3% (12/90) and 11.1% (20/180), respectively, which results in a case-phenotype. The training contingency table then has the property that (NTP+NFN) = (NFP+NTN) = 1.0. The resulting contingency table from the testing data in an N-fold cross-validation has (NTP+NFN) = (NFP+NTN) = 1/(N-1), so that the training contingency table is still (N-1) times larger than the testing contingency table.

As described, scoring functions 6 (Gini Index) and 7 (Absolute Probability Difference) are based on the distribution of all samples in the genotype-phenotype table. The scoring for metric 6 uses the Gini Index formula, which is used in CART decision trees [12]. The Gini Index for a given genotype (k) is GINI(k) (Table 2). P(j|k) is the relative frequency of class j (i.e. case or control) in genotype k. If there are a total of n samples in the study, and n_k _samples are have genotype k, the Gini Index for the distribution of the table is given by the GINIsplit formula. A better separation of cases and controls by a genotype combination results in a reduction of the value of GINIsplit. In order to maximize the formula, scoring is based on GINIparent-GINIsplit, where GINIparent is the un-separated state with all subjects in the same cell. Scoring function 7 is the sum of the absolute difference in probabilities of finding the cases and controls across all genotypes. Since both scoring functions 6 and 7 do not require N-fold cross-validation or formation of the contingency table, these are well suited for studies with a large number of SNPs or features.

**Table 2:** Number of times interacting alleles were observed as highest scoring model (rank = 1) or second (rank = 2) using PIA 2.0^a ^for 2-SNP interactions using balanced simulated data sets.

		Scoring Function							
Model Number^b^	Rank	%Correct	Sensitivity +Specificity	PPV+NPV	Risk Ratio	Odds Ratio	Gini Index	Probability Difference	Total (Overall)^c^
55	1	66	66	50	36	50	83	67	79
	2	4	4	14	11	14	10	15	6
56	1	55	55	46	33	46	84	58	68
	2	3	3	10	11	9	2	5	5
57	1	58	58	52	19	52	82	66	78
	2	7	7	10	12	10	8	7	9
58	1	88	88	76	58	76	96	89	92
	2	1	1	11	18	11	3	4	5
59	1	50	50	45	36	45	64	48	61
	2	13	13	13	16	12	10	14	11

^a ^Data were generated using cell counts to assign case versus control status (IFRACT = 0) and excluded the training data when scoring and running 10 10-fold cross-validations for functions 1–5 (ITRAIN = 0, FRACT = 0.1, NTIME = 10). Results including training data in scoring are presented in Additional file 1, Table S1.

^b ^Simulated data sets were described previously [14] and were obtained from Dr. Moore by request.

^c ^Total score is the summation over all scoring functions after linearly scaling the score for each individual function such that the top score is 50.0.

It should be noted that if the number of cases is equal to the number of controls, scoring metrics 1 (% Correct) and 2 (Sensitivity + Specificity) produce identical results. Therefore, if the number of samples, as opposed to fractional occupations, in the genotype-phenotype table is used for the assignment of phenotypes, all seven metrics are used. If fractional occupations are used, the first quality metric is dropped and only metrics 2 through 7 are used since metrics 1 and 2 will produce identical results.

PIA v. 2.0 stores the top 100 SNP combinations for each scoring metric used. The strength of examining multiple SNP combinations, as observed previously [5], is that while one model may perform best, the score of the top model may be only slightly higher than the models below it. In complex chronic diseases, it is likely that several genetic pathways are related to disease susceptibility. Therefore, selecting only the highest scoring model may result in reduced sensitivity of detecting interactions in multiple pathways. Once the PIA analysis is completed, the quality scores are linearly scaled so that the highest score has a value of 50. The scores are added for each SNP combination, resulting in a total score over all scoring functions. We theorized that averaging over all scoring functions would be superior to using any individual scoring function when examining SNP-SNP interactions since it would reduce the effect of false-positives identified by a single method or reduce the possible bias generated by any single method. SNP combinations are then ordered according to the total score. It is important to note that if there are fewer than 100 SNPs, there will be fewer than 100 1-SNP models.

The original version of PIA (v. 1.0) contained five different scoring functions, but only two were used in practice (% Wrong, which is analogous to % Correct in the current version, and Gini Index). The current version has incorporated several additional functions, but it is possible to select functions used in the original version. In addition to more methods of ranking SNP combinations, handling of ties (genotype combinations assigned to undetermined phenotypes), missing data, and combining testing and training data is modified in PIA v. 2.0. PIA v. 1.0 required that only one scoring function be used in a given run, while the current version ranks the SNP combinations for each of the seven scoring functions in a single run. In addition, overall ranks of SNP combinations are determined using the combined score across these seven functions by storing the top 100 SNP combinations for each scoring function and then linearly scaling their scores so that the best combination has a score of 50.0. The overall score for a given SNP combination is then the sum of the scores it receives across the seven functions.

PIA v. 2.0 lists the number of times SNPs and SNP-pairs (in SNP triplets or quartets, if they are examined) appear in the top 100 combinations for each scoring metric and overall, both as counts and using the scaled scores, as long as they appear more times than expected on average. When studying the complex 3-SNP and 4-SNP interactions, investigating the SNP-pairs that appear frequently in higher order combinations may provide some additional clues about combinations of genes with a role in disease risk. Moreover, by studying pairs, it is possible to examine interactions with a particular gene of interest, i.e. explore what additional genes or SNPs are often observed in combination with the gene/SNP of interest.

Finally, PIA v. 2.0 determines the number of times each gene pathway appears in the top 100 combinations if the user-defined pathway information for each gene is present in the allele file. Scores for each pathway are derived from the individual scores for each combination. Expected scores are based on random distribution of pathways among SNP combinations, accounting for the number of SNPs in each pathway within the data set. Observed and expected scores for each pathway are tabulated.

### Simulation data

Simulated data sets that model genetic interaction were created as described previously [14] and obtained from Dr. Moore. Each data model had 2 interacting alleles in the context of non-interacting SNPs (20 total SNPs for Balanced data sets, 10 for Imbalanced sets). Seventy different penetrance functions were generated with several different heritabilities and minor allele frequencies and probability models. For each of the seventy functions, 100 data sets were generated. Balanced data sets were available in population sizes of 400, 800, and 1600. Imbalanced data sets were created by randomly sampling cases and controls from within the larger simulated data sets to obtain the 1:2 or 1:4 case control ratios [14].

To demonstrate the ability of PIA to detect interactions, we examined SNP-pairs and determined the number of times the interacting alleles were observed as the highest or second highest scoring model across the 100 data sets. The percent of times the interacting alleles were correctly observed represents the power of PIA to detect interactions (or the efficiency under the alternative hypothesis). This approach was used previously to estimate power of MDR [14]. Our analysis was limited to the population size of 1600 because study populations less than 1600 are underpowered to detect gene*environment interaction. Our analysis was also limited to penetrance functions of heritability models of 0.01 and minor allele frequency (MAF) of 0.2, or models 55–59 in the Velez manuscript, where MDR had the lowest sensitivity at detecting the interacting SNPs [14]. The lower heritability assumes a weaker genetic effect on variation in phenotype. The low MAF is likely more representative of common genetic variation typically measured in population-based genetic epidemiology studies, and requires a more powerful tool for detection.

### Example data

PIA v. 2.0 was used to evaluate SNP combinations and interactions in a case-control study of colon cancer in a population 216 male cases and 255 male controls from the greater Baltimore area as described previously [5]. Genotyping data was available on 94 SNPs in 7 user-defined pathways (1-apoptosis, 2-inflammation, 3-DNA repair, 4-hormone metabolism, 5-metabolism, 6-angiogenesis, 7-other). The complete list of polymorphisms, rs numbers, and description of quality control for genotyping was reported previously [5]. All SNPs and assays may be found in the SNP500 database [15,16].

## Results

### Simulation results

Using the simulated data sets of balanced case:control ratios with a population size of 1600, heritability of 0.01 and MAF of 0.2, the highest scoring 2-SNP interactions were investigated using PIA v. 2.0. When only the testing data was used to construct the contingency table for the first five scoring functions, the interacting SNPs were identified as the highest total scoring 2-SNP model (average score over all scoring functions) in 76% of the balanced data sets (Table 2). The interacting SNPs were observed as either the 1^st ^or the 2^nd ^model in 82% of the data sets using the total scoring. Results were similar when the training data also contributed to the contingency table using either a leave-one out procedure or maximum likelihood procedure for the balanced data sets (Additional file 1). When using simulated data sets generated with a higher heritability ratio (0.3), the interacting SNPs were observed as the top model in 100% of the data sets (data not shown).

We examined the power of PIA v.2.0 to detect interactions over several scoring functions. The total score performed better than most of the other scoring functions, except for the Gini Index. The Gini Index was the highest performing function across the balanced data sets. The worst performing function was the Risk Ratio function. Note that the % Correct and Sensitivity + Specificity are identical in the situation with the same number of cases and controls.

The commonly observed interacting pairs in 3-SNP models were examined in the balanced data sets using PIA v. 2.0 (Table 3). The interacting alleles were present in many of the top scoring 3-SNP models. They were observed as the highest rank in the top 10 triplets in 72% of the data sets. The interacting SNPs were observed in the 1^st ^or 2^nd ^ranking of pairs in the triplets in 80% of the data sets.

**Table 3:** Number of times interacting alleles were observed as highest (rank = 1) or second highest (rank = 2) pairs in the top 10 triplets using PIA 2.0^a ^for 3-SNP interactions using balanced simulated data sets

		Scoring Function							
Model Number^b^	Rank	%Correct	Sensitivity +Specificity	PPV+NPV	Risk Ratio	Odds Ratio	Gini Index	Probability Difference	Total (Overall)^c^
55	1	73	73	79	58	79	81	67	63
	2	9	9	5	9	5	7	15	12
56	1	61	61	62	50	62	79	51	68
	2	10	10	6	8	6	6	10	8
57	1	72	72	71	47	71	78	55	75
	2	7	7	10	12	10	8	15	7
58	1	92	92	90	85	90	96	86	92
	2	3	3	2	4	2	2	4	3
59	1	57	57	55	52	55	61	42	60
	2	10	10	10	8	10	10	9	10

^a ^Data were generated using cell counts to assign case versus control status (IFRACT = 0) and excluded the training data when scoring and running 10 10-fold cross-validations for functions 1–5 (ITRAIN = 0, FRACT = 0.1, NTIME = 10). Results including training data in scoring are presented in Additional file 1, Table S1.

^b ^Simulated data sets were described previously [14] and were obtained from Dr. Moore by request.

^c ^Total score is the summation over all scoring functions after linearly scaling the score for each individual function such that the top score is 50.0.

To deal with imbalanced data, PIA v. 2.0 includes the option of using fractional occupations for scoring functions 1–5. PIA v. 2.0 was applied to imbalanced data sets with case:control ratios of 1:2 and 1:4 and 10 total SNPs. When using the option of fractional occupations (IFRACT = 1), scoring functions % Correct (1) and Sensitivity + Specificity (2) reduce to the same function. Therefore, for the analysis of the imbalanced data only the Sensitivity + Specificity function was included. Using fractional occupations, PIA v. 2.0 was able to detect the interacting SNP as the 1^st ^ranking 2-SNP model in 80% (1:2) and 68% (1:4) of the data sets (Table 4). The interacting SNPs were in the 1^st ^or 2^nd ^rank of 2-SNP models in 88% (1:2) or 77% (1:4) of the data sets. Results were similar when using both the training and testing data in scoring by using either a leave-one out procedure or maximum likelihood procedure for the imbalanced data sets (Additional file 1). As in the balanced datasets, the interacting SNP-pair was also frequently observed in many of the highest scoring 3-SNP models (Additional file 1). In contrast to the results obtained on the imbalanced data sets using fractional occupations, using cell counts alone with model 55 resulted in the interacting SNPs being observed 13 times (1^st ^rank) and 46 times (2^nd ^rank) in the 2-SNP models.

**Table 4:** Number of times interacting alleles were observed as highest scoring model (rank = 1) or second (rank = 2) using PIA 2.0^a ^for 2-SNP interactions using imbalanced simulated data sets

			Scoring Function						
Model Number^b^	Case:Control Ratio	Rank	Sensitivity +Specificity	PPV+NPV	Risk Ratio	Odds Ratio	Gini Index	Probability Difference	Total (Overall)^c^
55	1:2	1	72	72	69	72	81	74	82
		2	6	7	7	7	9	8	6
	1:4	1	56	50	50	50	61	63	67
		2	9	13	10	13	11	11	7
56	1:2	1	69	67	59	67	83	73	79
		2	8	10	10	10	11	9	10
	1:4	1	45	43	38	43	62	52	62
		2	10	11	12	11	15	12	11
57	1:2	1	63	62	45	62	82	69	76
		2	6	7	12	7	7	14	8
	1:4	1	51	49	34	49	67	53	66
		2	10	8	16	8	10	14	9
58	1:2	1	87	86	79	86	96	92	95
		2	7	5	8	5	4	4	4
	1:4	1	73	70	66	69	84	79	84
		2	8	8	15	9	10	8	7
59	1:2	1	64	61	62	61	72	72	70
		2	6	9	12	9	7	7	10
	1:4	1	54	50	45	50	48	60	61
		2	8	10	15	10	19	8	13

^a ^Data were generated using fractional occupations to assign case versus control status (IFRACT = 1) and excluded the training data when scoring and running 10 10-fold cross-validations for functions 1–5 (ITRAIN = 0, FRACT = 0.1, NTIME = 10). Training data is included in Additional file 1, Table S2.

^b ^Simulated data sets were described previously [14] and were obtained from Dr. Moore by request.

^c ^Total score is the summation over all scoring functions after linearly scaling the score for each individual function such that the top score is 50.0.

When comparing the performance of the different scoring functions at predicting the interacting SNPs in the imbalanced data sets, as with the balanced data, the Gini Index was the most powerful function at scoring interactions and the total scoring function performed better than all the other functions (Table 4, Additional file 1). The frequency of detecting the interacting SNPs was almost the same using the Gini Index, or the total scoring function. However, in some data sets, the total scoring function detected the interacting SNPs slightly better than the Gini Index. Unlike scoring functions 1–5, the Gini Index uses the distribution in the entire data set and does not require cross-validation. Given that the interacting SNPs were most frequently detected using the Gini Index, we examined the detection of the interacting SNPs using only a single run of the data, instead of using the 10 cycles of 10-fold cross-validation. Results were similar using only a single run of the data (data not shown).

### Example application

The top scoring SNP combinations for all combinations of containing 1–4 SNPs associated with colon cancer are shown (Table 5). The models with highest scores, or best predictors, were similar across the scoring functions. For example, the top model, with the highest average score, for the 2-SNP combinations was *CASP8_03 *plus *GSTT1_02*. This combination was the highest scoring combination for 3/7 of the scoring functions. The association of this combination with colon cancer using several different methods of scoring provides further evidence for the importance of this combination of SNPs in colon cancer risk. *IL1B_01 *plus *IL1B_03 *was the highest-ranking 2-SNP combination using only the Gini Index scoring function.

**Table 5:** Highest scoring SNP combinations associated with colon cancer using PIA v. 2.0^a^

**Number of SNPs**	**Scoring Function**							**Total (Overall)**^b^
	**%Correct**	**Sensitivity +Specificity**	**PPV+NPV**	**Risk Ratio**	**Odds Ratio**	**Gini Index**	**Probability Difference**	
1	GSTT1_02	GSTT1_02	PTGS2_11	PTGS2_11	PTGS2_11	IL4_01	GSTT1_02	PTGS2_11
2	CASP8_03	CASP8_03	TGFB1_02	TGFB1_02	TGFB1_02	IL1B_01	CASP8_03	IL1B_01
	GSTT1_02	GSTT1_02	PTGS2_11	PTGS2_11	PTGS2_11	IL1B_03	GSTT1_02	IL1B_03
3	MTRR_01	ESR1_03	TGFB1_02	TGFB1_02	TGFB1_02	IL1B_01	IL4_01	IL4_01
	IL1B_03	GPX1_03	CDC25A_02	CDC25A_02	CDC25A_02	IL1B_03	MTRR_01	MTRR_01
	SOD2_01	GSTT1_02	PTGS2_11	PTGS2_11	PTGS2_11	SOD2_01	DIO1_04	DIO1_04
4	race	race	CHEK1_02	CHEK1_02	CHEK1_02	WRN_03	IL4R_02	ESR1_03
	IL1B_01	IL1B_01	TGFB1_02	TGFB1_02	TGFB1_02	IL5_02	SOD2_01	GPX1_06
	IL1B_03	IL1B_03	TNF_02	TNF_02	TNF_02	IL10_02	GSTT1_02	CYP19A1_06
	MTHFR_02	MTHFR_02	CDC25A_02	CDC25A_02	CDC25A_02	CYP19A1_06	CYP19A1_09	GSTT1_02

^a ^Data were generated using cell counts to assign case versus control status (IFRACT = 0) and excluded the training data when scoring and running 10 10-fold cross-validations for functions 1–5 (ITRAIN = 0, FRACT = 0.1, NTIME = 10).

^b ^Total score is the summation over all scoring functions after linearly scaling the score for each individual function such that the top score is 50.0.

When examining the top 10 most commonly observed SNP-pairs from among the 3-SNP combinations, several SNP pairs were observed more commonly than expected by chance, including *GSTT1_02 *with *CASP8_03 *(Table 6). If the top 100 3-SNP combinations were randomly selected, a given SNP pair should appear 0.470 times, or about 3.3 times across the seven scoring functions. Therefore, these SNP-pairs were observed more frequently than expected by chance. Interaction between *GSTT1_02 *with *CASP8_03*, observed previously [5], was more clearly demonstrated in PIA v. 2.0 by examining SNP pairs among triplets using several different scoring functions (Table 6).

**Table 6:** Top 10 most frequently observed SNP-pairs in the 100 highest scoring triplet SNP combinations associated with colon cancer^a^.

**SNP Pair**		**Frequency of SNP Pair Observed in Each Scoring Function and Overall**							
**SNP-1**	**SNP-2**	**%Correct**	**Sensitivity +Specificity**	**PPV+NPV**	**Risk Ratio**	**Odds Ratio**	**Gini Index**	**Probability Difference**	**Total (Overall)**
IL1B_01	IL1B_03	9	7	0	0	1	71	3	91
CASP8_03	GSTT1_02	20	21	4	0	4	0	3	52
CHEK1_02	TGFB1_02	0	0	14	17	14	0	0	45
MTRR_01	SOD2_01	9	9	3	0	3	4	11	39
CDC25A_02	PTGS2_11	0	0	10	12	10	0	0	32
CHEK1_02	CDC25A_02	0	0	10	11	10	0	0	31
CHEK1_02	PTGS2_11	0	0	8	8	8	0	0	24
CHEK1_02	ALOX5_07	0	0	7	7	7	0	0	21
IL4_01	XRCC1_1	4	7	3	0	3	1	2	20
MTRR_01	DIO1_04	3	3	1	0	1	3	9	20

^a ^Top 100 triplet SNP combinations were generated using cell counts to assign case versus control status (IFRACT = 0). The training data were excluded in scoring and running ten 10-fold cross-validations for functions 1–5 (ITRAIN = 0, FRACT = 0.1, NTIME = 10). If the SNPs were randomly assigned to the top 100 triplet models, a given pair is expected to be observed 3.3 times overall.

## Discussion

Molecular epidemiology is entering a new era owing to advancements in genotyping technology and annotation of variation in the human genome. Few established statistical and bioinformatics tools exist for studying complex interactions underlying common diseases such as cancer and cardiovascular diseases. There is no *a priori *way to determine which method would be best to identify complex interactions in experimental datasets with variable minor allele frequencies and unknown heritability. Therefore, we developed a method to explore complex interactions. PIA v. 2.0 incorporates several different scoring functions for cross-validation. PIA v. 2.0 also deals with imbalances between case and control ratios, a common design feature of case-control studies. Moreover, PIA v. 2.0 allows stratification to determine if particular combinations of SNPs are associated with a phenotype (i.e. case vs. control) only in a particular subgroup of individuals. Finally, PIA v. 2.0 allows examinations of associations of user-defined pathways (based on SNP or gene) with a particular phenotype.

In this study, we applied PIA v. 2.0 to simulated data sets with 2 interacting SNPs in the context of non-interacting SNPs for a total of 10 (imbalanced) or 20 (balanced) SNPs and examined the power of PIA v. 2.0 to detect the interacting SNPs. The genetic model for these data sets assumed a modest genetic association with phenotype (MAF of 0.2 and heritability of 0.01). PIA v. 2.0 detected the interacting SNPs as the highest-ranking model in 77% of the data sets, and using some models, detected the SNPs in over 90% of the data sets. These results indicate that PIA is a powerful tool for detecting interactions.

A variety of approaches for studying complex interactions exist [5,10,17-19], but clearly additional methods are needed [11]. Multiple approaches should be implemented when examining complex data to reduce the likelihood of false positive associations [11,12,18]. PIA v. 2.0 was designed incorporating several scoring functions in order that SNP-SNP interactions may be validated over several functions, and ranked according to a total score. Using the simulated data, the total scoring function in PIA v. 2.0 performed better than all of the scoring functions other than the Gini Index. In addition, the interacting SNPs were observed in the 1^st ^model using PIA v. 2.0 more frequently than when using MDR [14]. As shown with the example colon cancer data, *CASP8_03 *plus *GSTT1_02 *was the highest scoring 2-SNP combination overall, while *IL1B_01 *plus *IL1B_03 *was the highest-ranking 2-SNP combination using only the Gini Index scoring function, indicating that each function may have strengths in different contexts or datasets. Of note, *IL1B_01 *and *IL1B_03 *are in linkage disequilibrium [5].

The interacting SNPs in the simulated data sets were most frequently detected using the Gini Index scoring function, compared with the total score. The reason for this is unclear, but may be due to the fact that the Gini Index uses the entire data set in scoring, instead of dividing the population into testing and training data. It is possible, with the balanced data the scoring is biased based on the counting of the same function twice. In the balanced data sets, the number of cases and controls were the same. In this situation, the % Correct and Sensitivity scoring functions reduce to the same formula. However, even with the imbalanced data, where fractional occupations are used and the % Correct function is excluded for the scoring, the Gini Index performed better than the total score.

In addition to examining the highest scoring SNP models, to increase sensitivity, we suggest that several of the top scoring models should be investigated [5]. The difference in score between the 1^st^ranking and 2^nd ^ranking SNP models may be modest. Only studying the highest scoring model may result in missing relevant associations [5]. For example, when reviewing the results of the 2-SNP interactions, the evidence for the *IL1B_01 *plus *IL1B_03 *(1^st ^scoring 2-SNP model) and the *GSTT1_02 *plus *CASP8_03 *(2^nd ^highest scoring model, data not shown) interactions are similar, both were high scoring models using multiple scoring functions. In this report, using the simulated genetic data, the interacting SNPs were frequently observed in the 2^nd ^ranking SNP models (~10% of data sets).

In addition to exploring the 1–4 SNP combinations most strongly associated with outcome, PIA v. 2.0 allows the user to examine the most commonly occurring SNP pairs among the top scoring 3-SNP models. The interacting SNPs were frequently detected as pairs when using PIA to examine the 3-SNP models using the simulated genetic data. Therefore, using PIA v. 2.0, there are multiple approaches to exploring 2-SNP interactions. It is unclear at present, which approach is the more sensitive approach. However, it should be noted that the data sets used in this paper, while appropriate for testing the ability of PIA v. 2.0 to detect interacting SNPs, might not accurately represent the situation in complex diseases. In complex diseases, multiple genes interact in complex pathways, as opposed to only 2 interacting SNPs in the context of non-interacting SNPs – i.e. in real data there are likely many competing interactions and alternative pathways. In this context, if an investigator is interested in 2-SNP interactions, it may be more appropriate to study the commonly occurring pairs in the 3-SNP models. Until the better approach is determined, we suggest that both looking at the 2-SNP models and the pairs observed in the 3-SNP models is the optimal approach.

Dealing with imbalances in case:control ratios is a challenge using multi-locus approaches for examining gene*gene interactions [14]. Using fractional occupations, PIA v. 2.0 detected the interacting SNPs in 70–80% of the imbalanced data sets (1:2 and 1:4 ratios), if only considering the 1^st ^ranking 2-SNP models. In contrast, if cell counts were used for imbalanced data, PIA v. 2.0 performed poorly. As a result, using the option of fractional occupations, PIA v. 2.0 may be applied to imbalanced case-control data to explore interactions.

Overall, PIA v. 2.0 is a robust method for detecting SNP-SNP interactions, as exemplified by the ability of PIA to detect the two interacting SNPs in the context of non-interacting SNPs using the simulated genetic data. PIA v. 2.0 detected the interacting SNPs more frequently than MDR using both the balanced and imbalanced data sets [14]. Moreover, PIA was more efficient at detecting interactions than exploring interactions (one at a time) using traditional methods. Different methods may be stronger in different contexts or datasets. It should be noted that PIA v. 2.0 was designed to be highly sensitive at detecting modest interactions. Therefore, even in absence of interacting SNPs, PIA v. 2.0 will identify top scoring models. To reduce false positive associations, users should carefully examine output produced by PIA v. 2.0 for consistent associations by investigating the detection of interactions using multiple scoring functions and the top pairs in 3-SNP or 4-SNP models and replicate observed associations using alternative methods (including traditional approaches). Furthermore, the results generated using simulated data should be interpreted with caution. PIA v. 2.0, and other methods for examining complex interactions, are likely less powerful in the context of competing interactions or alternative pathways. Further research is needed to evaluate PIA v. 2.0 in this context.

Several studies examined association of genetic variation with disease using large-scale multi-locus approaches. Previously, PIA was implemented in a study of colon cancer to examine complex interactions using 94 SNPs in 67 genes [5]. CART decision trees were used in a study of 16 SNPs in breast cancer [20], and 44 SNPs in bladder cancer [21]. Multifactor Dimensionality Reduction (MDR) was used to investigate 51 SNPs in 36 genes in multiple sclerosis [22], 36 gene variants in a nested case-control study within the EPIC cohort to study of bladder cancer, lung cancer and myeloid leukemia [23], and seven DNA repair SNPs in bladder cancer [24]. Another study explored the association of 16 genetic variants in 11 genes with Crohn's Disease using regularized least squares [19]. All of these studies observed complex genetic interactions associated with disease.

PIA v. 2.0 incorporates some aspects of the more common approaches implemented in other studies of complex genetic interaction, CART decision trees and MDR. The Gini Index, one of the PIA v. 2.0 scoring functions, is used for splitting branches in CART decision trees [25]. PIA v. 2.0 uses a form of dimensionality reduction, like in MDR, in the assignment of the genotype-phenotype table in Figure 1. The % Correct scoring function is similar to the scoring function used in MDR [10]. A reason for using a percent score (as in PIA v. 2.0), compared to the number correctly classified (as in MDR) for scoring of genotype combinations, is that it accounts for different sized populations when scoring cells; i.e. if a phenotype has a smaller population size but 50% of samples are still misclassified, the score is the same as if a phenotype had a larger populations size with 50% misclassification.

With the advent of genome wide association studies (GWAS), it is possible to genotype over 500,000 SNPs on a single individual. In complex diseases, there are likely many genes that interact in pathways that are related to disease susceptibility. As a result, in GWAS, there is an interest in exploring complex gene*gene interactions. Investigating complex gene*gene interactions is a challenge due to the computational time required with such a large amount of genotyping data. We observed, using PIA v. 2.0, that a single run of cross-validation was powerful at detecting the 2-SNP interactions similar to 10 rounds of 10-fold cross-validation. Further, the Gini Index and the Absolute Probability Difference functions, which both only implement a single run of the data, were robust at detecting the 2-SNP interactions. PIA also allows for the incorporation of user-defined pathways in the analysis of SNP interactions, which may be used to explore the association of global pathways, or gene ontologies with disease outcome. Therefore, while PIA v. 2.0 currently can only be used for up to 1400 SNPs, using a single run of the data or scoring functions 5 and 6, are a possible approach to be implemented to reduce computational time and may eventually be applied to analysis of GWAS.

In this paper, we describe a new method for exploring genetic interactions, but some of the limitations should be considered. In classifying genotype combinations associated with disease, PIA, as other dimensionality reduction methods, effectively dichotomizes exposure as "low" or "high" risk. Such a simplification of genotype combinations results in a loss of information, because in reality each SNP combination may be associated with levels of risk. In addition, PIA v. 2.0 is not equipped for continuous variables, such as age or years of smoking exposure. These types of variables may only be analyzed using PIA if split into a maximum of five categories. While PIA v. 2.0 is more powerful that traditional methods, when studying higher order interactions, associations become less stable due to the reduced number of individuals in each cell. Therefore, PIA does not eliminate the need to conduct studies of large sample sizes and to confirm findings with more traditional statistical methodologies.

## Conclusion

In conclusion, when evaluating a large number of genetic factors associated with disease, a strategy for focusing on only select complex interactions is more efficient and results in fewer comparisons. PIA v. 2.0 is a useful tool for exploring these interactions, generating hypotheses for gene*gene or gene*environment interaction, which may be investigated further using other statistical methods and in replication study populations.

## Availability and requirements

PIA v. 2.0 must be implemented on a Windows PC using the command window. The following documents and programs for use of PIA v. 2.0 in the zip file PIA2-distribution.zip: PIA2_Guide.doc, the user's manual; PIA2_examples.doc, step by step instructions with example data; test.csv, example data; csv2pia2.exe, converts csv files to pia files; csv2pia2.list, program that controls the conversion of the csv file to run csv2pia2.exe; pia2.exe, executable PIA v 2.0 program; pia2.list, control file to direct pia2.exe; pia2html.exe, program to generate PIA output in html format; pia2html.list, program to run pia2html.exe. PIA is available for download from the following website .

## Implementation

PIA v. 2.0 was written in Fortran and is available for implementation on PC platforms from the following website [26] with detailed descriptions and examples. Two files are required to run PIA v. 2.0, an allele file that contains the data to analyze and a control file that determines the type of analysis to be conducted. This latter file must be named pia2.list. The allele file includes all genotypes (SNPs, haplotypes), exposures, phenotypes (case vs. control; long vs. short survivorship) and potential confounders, and optional pathway numbers (if a user wants to investigate pathways). All variables must be categorical, with at most five categories for each variable numbered as any set of non-negative sequential integers. The control file specifies which allele file to be analyzed and the parameters to be implemented (Additional file 1, Table S6). PIA v. 2.0 may be used to analyze up to 1400 SNPs (or factors), in up to 1000 subjects, and up to 4^th ^order interactions (or combinations of factors associated with phenotype).

## Authors' contributions

LM and JG contributed to the design of the method and interpretation of data; BL developed method, wrote software for PIA and aided in interpretation; SC developed and conducted genotyping assays; CH conceived of the study. All authors read and approved the final manuscript.

## Supplementary Material

Additional file 1 Supplemental Tables. Simulation results applying different options in PIA 2.0 and parameter descriptions. Click here for file

## References

[B1] Chen YC, Hunter DJ (2005). Molecular epidemiology of cancer. CA Cancer J Clin.

[B2] Balmain A, Gray J, Ponder B (2003). The genetics and genomics of cancer. Nat Genet.

[B3] Ioannidis JP, Ntzani EE, Trikalinos TA, Contopoulos-Ioannidis DG (2001). Replication validity of genetic association studies. Nat Genet.

[B4] Lohmueller KE, Pearce CL, Pike M, Lander ES, Hirschhorn JN (2003). Meta-analysis of genetic association studies supports a contribution of common variants to susceptibility to common disease. Nat Genet.

[B5] Goodman JE, Mechanic LE, Luke BT, Ambs S, Chanock S, Harris CC (2006). Exploring SNP-SNP interactions and colon cancer risk using polymorphism interaction analysis. Int J Cancer.

[B6] Moore JH (2003). The ubiquitous nature of epistasis in determining susceptibility to common human diseases. Hum Hered.

[B7] Chanock SJ, Manolio T, Boehnke M, Boerwinkle E, Hunter DJ, Thomas G, Hirschhorn JN, Abecasis G, Altshuler D, Bailey-Wilson JE, Brooks LD, Cardon LR, Daly M, Donnelly P, Fraumeni JF, Freimer NB, Gerhard DS, Gunter C, Guttmacher AE, Guyer MS, Harris EL, Hoh J, Hoover R, Kong CA, Merikangas KR, Morton CC, Palmer LJ, Phimister EG, Rice JP, Roberts J, Rotimi C, Tucker MA, Vogan KJ, Wacholder S, Wijsman EM, Winn DM, Collins FS (2007). Replicating genotype-phenotype associations. Nature.

[B8] Colhoun HM, McKeigue PM, Davey SG (2003). Problems of reporting genetic associations with complex outcomes. Lancet.

[B9] Hahn LW, Ritchie MD, Moore JH (2003). Multifactor dimensionality reduction software for detecting gene-gene and gene-environment interactions. Bioinformatics.

[B10] Ritchie MD, Hahn LW, Roodi N, Bailey LR, Dupont WD, Parl FF, Moore JH (2001). Multifactor-dimensionality reduction reveals high-order interactions among estrogen-metabolism genes in sporadic breast cancer. Am J Hum Genet.

[B11] Thornton-Wells TA, Moore JH, Haines JL (2004). Genetics, statistics and human disease: analytical retooling for complexity. Trends Genet.

[B12] Heidema AG, Boer JM, Nagelkerke N, Mariman EC, van der ADL, Feskens EJ (2006). The challenge for genetic epidemiologists: how to analyze large numbers of SNPs in relation to complex diseases. BMC Genet.

[B13] Ritchie MD, Hahn LW, Moore JH (2003). Power of multifactor dimensionality reduction for detecting gene-gene interactions in the presence of genotyping error, missing data, phenocopy, and genetic heterogeneity. Genet Epidemiol.

[B14] Velez DR, White BC, Motsinger AA, Bush WS, Ritchie MD, Williams SM, Moore JH (2007). A balanced accuracy function for epistasis modeling in imbalanced datasets using multifactor dimensionality reduction. Genet Epidemiol.

[B15] Packer BR, Yeager M, Burdett L, Welch R, Beerman M, Qi L, Sicotte H, Staats B, Acharya M, Crenshaw A, Eckert A, Puri V, Gerhard DS, Chanock SJ (2006). SNP500Cancer: a public resource for sequence validation, assay development, and frequency analysis for genetic variation in candidate genes. Nucleic Acids Res.

[B16] (2008). http://snp500cancer.nci.nih.gov/home_1.cfm.

[B17] Chatterjee N, Kalaylioglu Z, Moslehi R, Peters U, Wacholder S (2006). Powerful multilocus tests of genetic association in the presence of gene-gene and gene-environment interactions. Am J Hum Genet.

[B18] Coffey CS, Hebert PR, Ritchie MD, Krumholz HM, Gaziano JM, Ridker PM, Brown NJ, Vaughan DE, Moore JH (2004). An application of conditional logistic regression and multifactor dimensionality reduction for detecting gene-gene interactions on risk of myocardial infarction: the importance of model validation. BMC Bioinformatics.

[B19] D'Addabbo A, Latiano A, Palmieri O, Maglietta R, Annese V, Ancona N (2007). Regularized Least Squares Classifiers may Predict Crohn's Disease from Profiles of Single Nucleotide Polymorphisms. Ann Hum Genet.

[B20] Gerger A, Langsenlehner U, Renner W, Weitzer W, Eder T, Yazdani-Biuki B, Hofmann G, Samonigg H, Krippl P (2006). A multigenic approach to predict breast cancer risk. Breast Cancer Res Treat.

[B21] Wu X, Gu J, Grossman HB, Amos CI, Etzel C, Huang M, Zhang Q, Millikan RE, Lerner S, Dinney CP, Spitz MR (2006). Bladder cancer predisposition: a multigenic approach to DNA-repair and cell-cycle-control genes. Am J Hum Genet.

[B22] Motsinger AA, Brassat D, Caillier SJ, Erlich HA, Walker K, Steiner LL, Barcellos LF, Pericak-Vance MA, Schmidt S, Gregory S, Hauser SL, Haines JL, Oksenberg JR, Ritchie MD (2007). Complex gene-gene interactions in multiple sclerosis: a multifactorial approach reveals associations with inflammatory genes. Neurogenetics.

[B23] Manuguerra M, Matullo G, Veglia F, Autrup H, Dunning AM, Garte S, Gormally E, Malaveille C, Guarrera S, Polidoro S, Saletta F, Peluso M, Airoldi L, Overvad K, Raaschou-Nielsen O, Clavel-Chapelon F, Linseisen J, Boeing H, Trichopoulos D, Kalandidi A, Palli D, Krogh V, Tumino R, Panico S, Bueno-De-Mesquita HB, Peeters PH, Lund E, Pera G, Martinez C, Amiano P, Barricarte A, Tormo MJ, Quiros JR, Berglund G, Janzon L, Jarvholm B, Day NE, Allen NE, Saracci R, Kaaks R, Ferrari P, Riboli E, Vineis P (2007). Multi-factor dimensionality reduction applied to a large prospective investigation on gene-gene and gene-environment interactions. Carcinogenesis.

[B24] Andrew AS, Nelson HH, Kelsey KT, Moore JH, Meng AC, Casella DP, Tosteson TD, Schned AR, Karagas MR (2006). Concordance of multiple analytical approaches demonstrates a complex relationship between DNA repair gene SNPs, smoking and bladder cancer susceptibility. Carcinogenesis.

[B25] Breiman L, Friedman JH, Olshen RA, Stone CJ (1984). Classification and Regression Trees. Wadsworth. Belmont, CA.

[B26] (2008). http://www3.cancer.gov/intra/lhc/PIA2-distribution.zip.

