# Supplementary material for: Polymorphism Interaction Analysis (PIA): a method for investigating complex gene-gene interactions
Source: BMC Bioinformatics. 2008 Mar 6;9:146. doi: 10.1186/1471-2105-9-146 (PMC2335300; doi:10.1186/1471-2105-9-146)
Supplement: Additional file 1 — Supplemental Tables. Simulation results applying different options in PIA 2.0 and parameter descriptions. [file 1471-2105-9-146-S1.doc]

| Supplemental Table 1. Number of times interacting alleles were observed as highest scoring model (rank=1) or second (rank=2) using PIA 2.0^a^ for 2-SNP interactions using balanced simulated data sets | | | | | | | | | | |
| --- | --- | --- | --- | --- | --- | --- | --- | --- | --- | --- |
|  |  |  | Scoring Function | | | | | | |  |
| Model Number^b^ | Procedure | Rank | %Correct | Sensitivity +Specificity | PPV+NPV | Risk Ratio | Odds Ratio | Gini Index | Probability Difference | Total (Overall)^c^ |
| 55 | LOO | 1 | 63 | 63 | 50 | 38 | 50 | 83 | 67 | 78 |
|  |  | 2 | 6 | 6 | 15 | 13 | 15 | 10 | 15 | 6 |
|  | EM | 1 | 67 | 67 | 50 | 23 | 49 | 83 | 67 | 81 |
|  |  | 2 | 15 | 15 | 14 | 14 | 14 | 10 | 15 | 6 |
| 56 | LOO | 1 | 53 | 53 | 46 | 31 | 46 | 84 | 58 | 66 |
|  |  | 2 | 3 | 3 | 8 | 14 | 8 | 2 | 5 | 6 |
|  | EM | 1 | 57 | 57 | 43 | 21 | 42 | 84 | 58 | 71 |
|  |  | 2 | 6 | 6 | 10 | 11 | 11 | 2 | 5 | 4 |
| 57 | LOO | 1 | 57 | 57 | 54 | 21 | 54 | 82 | 66 | 73 |
|  |  | 2 | 3 | 3 | 8 | 11 | 8 | 8 | 7 | 11 |
|  | EM | 1 | 66 | 66 | 49 | 10 | 49 | 82 | 66 | 76 |
|  |  | 2 | 7 | 7 | 16 | 7 | 16 | 8 | 7 | 11 |
| 58 | LOO | 1 | 87 | 87 | 76 | 62 | 76 | 96 | 89 | 93 |
|  |  | 2 | 2 | 2 | 10 | 15 | 10 | 3 | 4 | 4 |
|  | EM | 1 | 89 | 89 | 72 | 46 | 72 | 96 | 89 | 91 |
|  |  | 2 | 3 | 3 | 12 | 19 | 11 | 3 | 4 | 5 |
| 59 | LOO | 1 | 48 | 48 | 45 | 40 | 44 | 64 | 48 | 61 |
|  |  | 2 | 12 | 12 | 10 | 17 | 11 | 10 | 14 | 11 |
|  | EM | 1 | 49 | 49 | 42 | 27 | 42 | 64 | 48 | 61 |
|  |  | 2 | 13 | 13 | 10 | 13 | 10 | 10 | 14 | 8 |
| ^a^ Data were generated using cell counts to assign case versus control status (IFRACT = 0) and included the training data  when scoring and running 10 10-fold cross-validations for functions 1-5 (ITRAIN=1, FRACT=0.1, NTIME=10). Training data is included using either a leave-one out procedure (LOO, LOOTR=1) or the maximum likelihood (EM, LOOTR=0). | | | | | | | | | | |
| ^b^ Simulated data sets were described previously (Valez et al., 2007) and were obtained from Dr. Moore by request. | | | | | | | | | |  |
| ^c^ Total score is the summation over all scoring functions after linearly scaling the score for each individual function such  that the top score is 50.0. | | | | | | | | | | |

| Supplemental Table 3. Number of times interacting alleles were observed as highest (rank=1) or second highest (rank =2) pairs in the top 10 triplets using PIA 2.0^a^ for 3-SNP interactions using imbalanced simulated data sets | | | | | | | | | |  |
| --- | --- | --- | --- | --- | --- | --- | --- | --- | --- | --- |
|  |  |  | Scoring Function | | | | | |  |  |
| Model Number^b^ | Case:Control Ratio | Rank | Sensitivity +Specificity | PPV+NPV | Risk Ratio | Odds Ratio | Gini Index | Probability Difference | Total (Overall)^c^ |  |
| 55 | 1:2 | 1 | 71 | 73 | 70 | 73 | 84 | 79 | 82 |  |
|  |  | 2 | 13 | 10 | 9 | 10 | 6 | 9 | 9 |  |
|  | 1:4 | 1 | 51 | 49 | 54 | 49 | 66 | 62 | 66 |  |
|  |  | 2 | 9 | 18 | 12 | 18 | 9 | 10 | 9 |  |
| 56 | 1:2 | 1 | 70 | 70 | 71 | 70 | 81 | 75 | 83 |  |
|  |  | 2 | 11 | 10 | 7 | 10 | 10 | 12 | 6 |  |
|  | 1:4 | 1 | 52 | 53 | 51 | 53 | 66 | 53 | 60 |  |
|  |  | 2 | 17 | 14 | 9 | 14 | 12 | 14 | 14 |  |
| 57 | 1:2 | 1 | 72 | 73 | 64 | 73 | 76 | 75 | 77 |  |
|  |  | 2 | 9 | 8 | 16 | 8 | 8 | 10 | 10 |  |
|  | 1:4 | 1 | 54 | 56 | 54 | 56 | 58 | 58 | 62 |  |
|  |  | 2 | 16 | 15 | 11 | 15 | 6 | 16 | 11 |  |
| 58 | 1:2 | 1 | 89 | 91 | 92 | 91 | 96 | 94 | 96 |  |
|  |  | 2 | 7 | 5 | 4 | 5 | 3 | 4 | 4 |  |
|  | 1:4 | 1 | 77 | 79 | 79 | 79 | 82 | 84 | 85 |  |
|  |  | 2 | 8 | 5 | 9 | 5 | 11 | 7 | 7 |  |
| 59 | 1:2 | 1 | 66 | 67 | 69 | 67 | 70 | 72 | 78 |  |
|  |  | 2 | 9 | 8 | 8 | 8 | 10 | 6 | 5 |  |
|  | 1:4 | 1 | 61 | 59 | 59 | 59 | 47 | 61 | 64 |  |
|  |  | 2 | 4 | 6 | 9 | 6 | 18 | 8 | 5 |  |
| ^a^ Data were generated using fractional occupations to assign case versus control status (IFRACT = 1) and excluded the training data when scoring and running 10 10-fold cross-validations for functions 1-5 (ITRAIN=0, FRACT=0.1, NTIME=10). Training data is included in Supplemental Table 2. | | | | | | | | | |  |
| ^b^ Simulated data sets were described previously (Valez et al., 2007) and were obtained from Dr. Moore by request. | | | | | | | | |  |  |
| ^c^ Total score is the summation over all scoring functions after linearly scaling the score for each individual function such  that the top score is 50.0. | | | | | | | | | | |

Supplemental Table 6. Descriptive list of PIA v. 2.0 parameters ^a^

| **Parameter name** | **Example value** | **Parameter description** |
| --- | --- | --- |
| NTERM | 2 | Number of SNPs/Factors to examine in combination |
| NSIFT | 1 | If NSIFT > 0, “Sift” or Stratifies data on factor |
| SIFT(I), SIFTV(I)  I=1,NSIFT | 2 1 | If NSIFT > 0, SIFT(I) is the number of the feature to sift. If SIFTV(I)=J, then the sample must have a value of J for feature SIFT(I); if SIFTV(I)=-J, samples can have any value but J; if SIFTV(I)=9, any values are allowed for SIFT(I), but this feature is excluded from the analysis |
| HPATH | 0 | Pathway numbers (0=absent, 1=present) |
| NTOP | 20 | Number of best combinations to report in detail in output |
| LOGIT | 0 | Reports intermediate results to file pia-log.out (1=report, 0=don’t report) |
| SEED | 12054789.0 | 8-byte integer (given as a floating-point number) to seed the random number generator |
| FRACT | 0.1 | Decimal fraction to be removed for N-fold cross-validation (0.1=10%=10-fold cross-validation) |
| RATSL | 1.7 | If maximum (cases,controls)/min (cases,controls) > RATSL for a particular combination, the combination is excluded from the analysis |
| NTIME | 10 | The number of times samples are scrambled and then run using N-fold cross-validation |
| IFRACT | 1 | Number of cases and controls (0) or fraction (1) which determines if a cell is “case” or “control” |
| ITRAIN | 0 | Training set does (1) or does not (0) contribute to the contingency table |
| LOOTR | 1 | If LOOTR=1, a leave-one-out procedure is used to construct the contingency table for the training data; LOOTR=0, a maximum likelihood procedure is used for the training data; LOOTR=-1, the procedure used in Version 1 [5] is used for both the training and testing data.^a^ |

^a^ To obtain the results outlined in [6], one should use IFRACT=0, ITRAIN=1 and LOOTR=-1. The %Wrong results will be listed under Scoring Function 2, and the PIA v. 1.0 %Wrong score is 2.0-(Sensitivity+Specificity score). The Gini results for PIA v. 1.0 are listed under Scoring Function 6 with the PIA v. 1.0 Gini(split) values equal to 1.0-Gini(index).
